# Multifoci Bone Tuberculosis and Lymphadenitis in Mediastinum Mimics Malignancy on FDG-PET/CT: A Case Report

**DOI:** 10.4274/Mirt.145

**Published:** 2014-02-05

**Authors:** Nalan Alan Selçuk, Ayşen Fenercioğlu, Hatem Hakan Selçuk, Çağatay Uluçay, Esin Yencilek

**Affiliations:** 1 Yeditepe University Hospital, Department of Nuclear Medicine, İstanbul, Turkey; 2 Yeditepe University Hospital, Department of Family Medicine, İstanbul, Turkey; 3 Bakırköy State Hospital, Department of Radiology, İstanbul, Turkey; 4 Yeditepe University Hospital, Department of Orthopedic Surgery, Istanbul, Turkey; 5 Haydarpaşa Numune Training and Research Hospital, Department of Radiology, Istanbul, Turkey

**Keywords:** Positron-emission tomography, 18FDG, bone tuberculosis, tumors, diagnosis

## Abstract

Positron Emission Tomography with 2-deoxy-[F-18]-fluoro-D-glucose (FDG-PET) has become a reliable diagnostic tool in clinical practice similar to Magnetic Resonance (MR) imaging and Computed Tomography (CT). FDG-PET has especially been used to differentiate malignant from benign lesions, and for staging and follow- up malignant tumors. However, FDG-PET has some pitfalls in cancer screening and FDG tracer accumulates at sites of infection and inflammation. Bone tuberculosis may be confused with malignant tumors of bone and its metastases, and can accumulate focally increased FDG in active period. We present a 60-year-old woman with lytic bone lesions and mediastinal hypermetabolic foci, initially suspected to be malignant by means of FDG-PET and the other imaging modalities; however, bone biopsy confirmed the diagnosis of bone tuberculosis.

**Conflict of interest:**None declared.

## Abstract (tr)

[F-18]-floro-deoksi-glikoz Pozitron Emisyon Tomografisi (FDG-PET), klinik uygulamalarımızda Manyetik Rezonans (MR) ve Bilgisayarlı Tomografi (BT) gibi güçlü bir tanı aracı haline gelmiştir. Özellikle lezyonların benign malign ayrımında, malign tümörlerin evrelendirilmesinde ve takibinde kullanılmaktadır. Ancak FDG-PET tümöre spesifik bir yöntem değildir ve infeksiyon/ inflamasyon gibi durumlarda da FDG birikimi görülebilir. Kemik tüberkülozu kemiğin malign tümörü veya metastazları ile karışabilir ve aktif dönemde ise artmış FDG tutulumu gösterebilir. Bu yazıda diğer görüntüleme tekniklerinde ve FDG-PET’de malign gibi gözüken kemikte litik lezyonları ve mediastende malignite düzeyinde hipermetabolik lenf adenopatileri mevcut olan 60 yaşındaki kadın olgu sunulmaktadır. İlk planda malignite lehine değerlendirilen olguda, tanıyı teyit amaçlı yapılan mediastenoskopik incelemede ve kemik biyopsisinde tüberkülozis tanısı kondu.

## INTRODUCTION

Tuberculosis (TB) is a chronic infectious disease caused by the tubercle bacilli. Although TB has been a well-known disease, it has not yet been completely eradicated throughout the world. New technologies and drugs may help the physicians fight against the resistant tubercle bacillus; however, TB remains to be major problem in developing countries (1). Its incidence is also high together with immune deficiencies such as HIV in developed countries (2). The prevalence of osseous and extra-pulmonary TB was reported to account for 1-15% of all TB infections (3,4). Involvement of bones and joints is secondary to pulmonary lesions. TB of bone usually appears two years or longer after the initial pulmonary disease (5). Bone and joint TB results from haematogenous spread from pulmonary, visceral or lymph node foci. Most of those lesions heal with immunity but in some cases bone and joint involvement may become manifest. Bone and joint tuberculosis is likely to occur in the spine, hip, knee, foot, elbow, wrist, hand, shoulder with haematogenous spread. Long bone tuberculosis is rather rarely encountered (6).

Positron Emission Tomography with 2-deoxy-[F-18]-fluoro-D-glucose (FDG-PET) plays an important role in differentiating benign from malignant tumors, staging and follow- up. However, FDG-PET is not a specific technique for cancer screening and FDG tracer accumulates at sites of infection and inflammation and it can also render some false positive results in differentiating tuberculosis from malignancy (7).

## CASE REPORT

**Figure 1:**
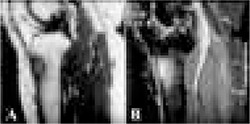
MR showed a 3x2 cm hypointense mass in T1 sequences (A) and heterogenic hyperintense lytic mass in SPIR sequences (B).

A 60-year-old woman presented to our hospital with a four- month history of increasing pain in the right knee. No difference was noted in pain character with motion and at rest. Pain was localized in medial tibial metaphysis. Local tenderness and painful arch of knee motion were observed without any tumoral tissue clinical inflammatory findings. She had no history of fatigue, cough, weight loss, diabetes, night sweats or of a malignancy. The blood count and CRP were within normal range. Sedimentation rate was elevated. A plain radiogram revealed a lytic lesion in proximal metaphysic of tibia. Computed tomography showed a lytic and exocentric lesion starting intramedullary and destructing the cortex with soft tissue extension, whereas MR delineated a 3x2 cm hypointense mass in T1 sequences and heterogenic hyperintense lytic mass with contrast enhancement in STIR sequences (Figure 1). Radiographically, the preliminary diagnosis was chondrosarcoma. The patient was referred to nuclear medicine department for confirmation of the preliminary diagnosis as well as for the investigation whether metastasis was present.

**Figure 2:**
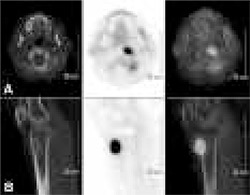
Selected CT, corresponding PET and fusion images from the leftside of the first cervical vertebra’s anterior arch and interarticular region (A), and proximal metaphysis of right tibia (B).

**Figure 3:**
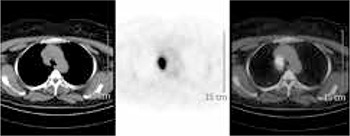
Selected transaxial CT, corresponding PET and fusion images of mediastinum showing FDG uptake in conglomerate nodules at right upper-lower paratracheal lymph nodes.

FDG-PET imaging with I.V. administration of 370 MBq FDG was performed following six hours of fasting while the patient had a serum glucose level of 92 mg/dl. We waited one hour for distribution of FDG in the body then the patient was imaged using PHILIPS Gemini Dual GS (Philips medical Systems, Cleveland UK) integrated PET/CT scanner with 2 slice CT. Whole body PET/CT showed increased FDG uptake at the left side of the anterior arch of the first cervical vertebra and at the interarticular region and at the proximal metaphysis of right tibia (Figure 2). Also in mediastinal slices, hypermetabolic conglomerated nodules measuring 3.5x3.0x4.0 cm were observed at right upper-lower paratracheal lymph nodes (Figure 3). The maximum standard uptake values (SUVmax) for vertebra, tibia and mediastinal lymph nodes were 8.4, 14.4 and 12.0, respectively. Neither FDG uptake nor fibrotic lesion was found in parenchyma of both the lungs. Normal physiological distribution of FDG was observed at the rest of the body. FDG-PET/CT results were initially evaluated as malignant lymphadenopathies at right lower paratracheal lymph nodes in the mediastinum, because there was increased FDG uptake in both lytic lesions located at the first cervical vertebra and proximal tibia.

Based on the results of imaging studies, oncology and chest diseases consultations were requestedt. The clinical manifestations, physical examination and blood tests were not in line with the imaging results. The patient underwent thoracic lymph node biopsy with mediastinoscopy and percutaneous bone biopsy from tibia under fluoroscopy. The pathologic examination confirmed the diagnosis of bone tuberculosis and lymph node involvement. The patient received chemotherapy with ethambutol, rifampicin and isoniacid for 6 months and recovered totally without needing any surgery. 

## LITERATURE REVIEW AND DISCUSSION

The musculoskeletal system is involved in only 1%-3% of cases with tuberculosis (8). The disease affects patients of all ages (other than the 1st year of life) and most frequently involves the spinal column, pelvis, hip and knee (9). The prevalence of osseous and extra-pulmonary TB has been reported to account for 1-15% of all TB infections (3,4). Bone and joint TB result from hematogenous spread from pulmonary, visceral or lymph node foci. Spine involvement has been reported in nearly 50% of bone TB (10). Long bone involvement is noted in only 10% of bone TB and metaphysical involvement is seen especially more frequently in adults. Similarly, we observed tibial metaphysical involvement in our patient. Epiphysial involvement can be seen particularly in children with active epiphysial growth plates (9).

Bone tuberculosis usually appears at least 2 years after pulmonary or visceral TB. In the light of chest X-ray and consultation with an expert in chest diseases, neither any mass nor any findings of physical examination did suggest pulmonary TB. The only positive result was broadening of the mediastinum on PA thorax x-ray. Only 33% of the patients with bone TB also have active pulmonary TB (2,11). Our patient did not have active pulmonary TB or TB history either.

FDG-PET has become a powerful diagnostic tool in the clinical practice similar to MR and CT. Nuclear medicine has began to take an important part in daily practice of orthopedics, thanks to the developments of medical technology. Not only does it inform us about malignancies using FDG-PET but it also provides valuable preoperative knowledge to traumatology, arhtroplasty and spine surgery (12). Unlike MR, FDG-PET can be performed when there is an implant or an artifact at the examination site (13). FDG-PET reliably confirms the diagnosis of TB (14,15). However, it lacks specificity in determining malignancy (7). FDG tracer accumulates at sites of infection and inflammation and it may yield false positive results in discriminating infection and inflammation from malignancy (4,16,17). For this reason, TB can easily be confused with malignancy due to characteristics of tuberculosis (9,18).

Taking late images generally facilitates differentiation of infection from malignancies. In infections, late images should indicate FDG washout from the tissue. However, washout was not observed in our case, even an increased FDG accumulation was noted.

In conclusion, any involvement of particularly the tibia by TB is rarely confronted and its symptoms may be similar to malignant tumors. Malignancy could be confused with TB by imaging studies including FDG-PET. FDG tracer accumulates at sites of infection and inflammation and it can render false positive results in infection and inflammation differentiating malignancy (2,5). Because of the characteristics of tuberculosis disease, TB and malignancy can easily be confused (4,7). We wanted to draw attention to the fact that increased FDG uptake may not always necessarily be an indicator of malignancy. In a patient with unknown primary malignancy, in the event that there is no clue of the primary focus on PET/CT, the metabolic activities of FDG foci has to be evaluated together with the structural changes on CT and the possibility of tuberculosis should be included in the diagnostic checklist.
